# Draft Genome Sequence of Staphylococcus epidermidis UMB7765, Isolated from the Urobiome of a Woman with Recurrent Urinary Tract Infection

**DOI:** 10.1128/MRA.00417-20

**Published:** 2020-05-14

**Authors:** Lucy Kemper, Taylor Miller-Ensminger, Adelina Voukadinova, Alan J. Wolfe, Catherine Putonti

**Affiliations:** a Department of Biology, Loyola University Chicago, Chicago, Illinois, USA; b Bioinformatics Program, Loyola University Chicago, Chicago, Illinois, USA; c Department of Microbiology and Immunology, Stritch School of Medicine, Loyola University Chicago, Maywood, Illinois, USA; d Department of Computer Science, Loyola University Chicago, Chicago, Illinois, USA; Georgia Institute of Technology

## Abstract

Staphylococcus epidermidis is a Gram-positive bacterium that is resistant to many antibiotics. Here, we present the 2.5-Mb draft genome of S. epidermidis UMB7765, isolated from a voided urine sample from a female with recurrent urinary tract infections.

## ANNOUNCEMENT

While Staphylococcus epidermidis primarily colonizes human skin (1), it has also been found to be a native member of the microbiota of other organs (2, 3). While often harmless, S. epidermidis is an opportunistic pathogen; it can cause serious infections of implanted medical devices (such as catheters and pacemakers) (4, 5). Treatment of S. epidermidis infections is complicated by the species’ often high levels of antibiotic resistance and ability to form biofilms (6, 7). Here, we present the genome sequence of S. epidermidis UMB7765, isolated from a voided urine sample from a woman with recurrent urinary tract infection (UTI). While S. epidermidis has on rare occasions been associated with UTIs (8), we do not have definitive evidence that this strain was the cause of UTI symptoms for this individual.

S. epidermidis UMB7765 was isolated using the expanded quantitative urine culture (EQUC) method (3) as part of a prior institutional review board (IRB)-approved study (University of California, San Diego, IRB no. 170077AW). The genus and species for this isolate were determined by matrix-assisted laser desorption ionization–time of flight (MALDI-TOF) mass spectrometry following protocols detailed previously (3). The isolate was stored at −80°C until sequencing. The freezer stock was first streaked onto a Columbia nalidixic acid (CNA) agar plate and incubated at 35°C with 5% CO_2_ for 24 h. Tryptone soy liquid medium was inoculated with a single colony from the plate and incubated overnight at 37°C. DNA extraction was done using the Qiagen DNeasy blood and tissue kit with the following modifications to the Gram-positive protocol: the bacteria were lysed using 230 μl of lysis buffer (180 μl of 20 mM Tris-Cl, 2 mM sodium EDTA, and 1.2% Triton X-100 and 50 μl of lysozyme) in step 2, and the incubation time in step 5 was altered to 10 min. The purified DNA was quantified using a Qubit fluorometer. DNA sequencing was done at the Microbial Genome Sequence Center at the University of Pittsburgh, where the DNA was fragmented using an Illumina tagmentation enzyme. Indices were attached using PCR and sequenced on the Illumina NextSeq 550 platform. Sequencing yielded 1,950,209 pairs of 150-bp reads. Unless otherwise noted, default parameters were used for all software tools. Reads were trimmed using Sickle v1.33 (https://github.com/najoshi/sickle). The reads were assembled using SPAdes v3.13.0 with the “only-assembler” option for k values of 55, 77, 99, and 127 (9). The genome coverage was calculated using BBMap v38.47 (https://sourceforge.net/projects/bbmap/). NCBI’s Prokaryotic Genome Annotation Pipeline (PGAP) v4.11 was used to annotate the publicly available genome sequences (10).

The draft genome sequence is 2,530,547 bp long, assembled into 68 contigs with an *N*_50_ value of 158,051 bp and 190× coverage. The GC content of the assembled genome is 31.93%, which is consistent with that of other publicly available S. epidermidis genomes. PGAP annotation includes 2,475 genes total, 2,361 encoding proteins, and 56 tRNAs. We analyzed the genome assembly using the Center for Genomic Epidemiology’s Web tool ResFinder v3.2 (11) and identified resistance genes for aminoglycosides, beta-lactams, fosfomycin, macrolides, and tetracycline. Frequently, antibiotics are prescribed for recurrent UTIs, and antibiotic resistance is likely (12). We can thus speculate that the multiple genes associated with antibiotic resistance encoded by S. epidermidis UMB7765 were acquired through repeated antibiotic exposure.

### Data availability.

This whole-genome shotgun project has been deposited in GenBank under the accession no. JAAUWD000000000. The version described in this paper is the first version, JAAUWD010000000. The raw sequencing reads have been deposited in the SRA under the accession no. SRR11441034.
